# Bayesian Coherence Analysis for Microcircuit Structure Learning

**DOI:** 10.1007/s12021-022-09608-0

**Published:** 2022-10-05

**Authors:** Rong Chen

**Affiliations:** Department of Diagnostic Radiology and Nuclear Medicine, University of Maryland School of Medicine, 100 N Greene, Baltimore, MD 21201 USA

**Keywords:** Microcircuit, Structure learning, Markov blanket, Bayesian network, Markov network

## Abstract

Functional microcircuits model the coordinated activity of neurons and play an important role in physiological computation and behaviors. Most existing methods to learn microcircuit structures are correlation-based and often generate dense microcircuits that cannot distinguish between direct and indirect association. We treat microcircuit structure learning as a Markov blanket discovery problem and propose Bayesian Coherence Analysis (BCA) which utilizes a Bayesian network architecture called Bayesian network with inverse-tree structure to efficiently and effectively detect Markov blankets for high-dimensional neural activity data. BCA achieved balanced sensitivity and specificity on simulated data. For the real-world anterior lateral motor cortex study, BCA identified microcircuit subtypes that predicted trial types with an accuracy of 0.92. BCA is a powerful method for microcircuit structure learning.

## Introduction

Coordinated neuronal firing has been reported in the cortical and subcortical regions (Harris et al., 2003; Uhlhaas et al., 2009; Oberto et al., 2022). Statistical associations among neural dynamics form a mesoscopic scale functional network (millimeter-to-micrometer resolution). Such mesoscopic scale networks are functional microcircuits (Chen, 2021). Functional microcircuits model coordinated activity of neurons firing in synchrony which has been proposed as the general substrate for a variety of physiological computation and behaviors  (Baeg et al., 2003; Dragoi & Buzsáki, 2006; Fujisawa et al., 2008).

Most existing studies examining functional microcircuits are based on correlation analysis and its variations. A typical correlation-based process is as follows: for *M* neurons, we first calculate $M(M-1)/2$ pairwise correlation coefficients, then use the false discovery rate for multiple comparison correction to remove weak associations. The major limitation of correlation-based microcircuit structure learning is that it often generates a dense brain graph.

We propose a method called Bayesian Coherence Analysis (BCA) to construct microcircuits for high-dimensional neural activity data. The foundation of BCA is Bayesian modeling for conditional independence. In BCA, the microcircuit structure learning problem is formulated as a Markov blanket discovery problem. BCA has the following characteristics: first, it generates a parsimonious model; second, it can efficiently generate a graphical model for high-dimensional data and has excellent scalability; third, it is a unified framework for both discrete data (binary spikes) and quantitative data (calcium imaging signal).

## Method

### Background

BCA is based on probabilistic graphical models (Koller & Friedman, 2009). Let $\mathcal {V} = \{X_1, ... , X_M\}$ denote a set of random variables taking values in a discrete or continuous state space $\Lambda$. Let $P_{\mathcal {V}}$ be a joint probability distribution on $\mathcal {V}$. $\mathbf {A}$ and $\mathbf {B}$ are conditionally independent given $\mathbf {C}$ if $P(\mathbf {A} \Vert \mathbf {B}, \mathbf {C}) = P(\mathbf {A} \Vert \mathbf {C})$, where $\mathbf {A, B, C}$ are mutually exclusive subsets of $\mathcal {V}$. A probabilistic graphical model encodes conditional independence of $P_{\mathcal {V}}$ into a graph structure $\mathcal {G} = \{ \mathcal {V}, \mathcal {E} \}$, where $\mathcal {V}$ is the node set and $\mathcal {E}$ is the edge (or link) set. The Markov Blanket of node $X_i$, denoted by $mb(X_i)$, is the minimal set of nodes that separates $X_i$ from the rest of the graph (Pearl, 1988). $X_i$ is conditionally independent of variables in $\mathcal {V} \setminus \{X_i, mb(X_i)\}$ given $mb(X_i)$. Nodes in $mb(X_i)$ consist of the minimal set (the most compact set) of variables that are jointly most predictive of $X_i$. For the variable selection problem, *mb*(*X*) is the optimal set to predict $X_i$.

Probabilistic graphical models represent joint distributions in concise forms. A compact representation can dramatically accelerate the inference process. There are mainly two types of probabilistic graphical models: Markov networks and Bayesian networks. Both can be used to study conditional independence among a set of random variables. In a Markov network, the structure $\mathcal {G}$ is an undirected graph and edges represent symmetric associations between nodes. If the graph topology is a lattice, a Markov network is also referred to as a Markov random field, which is widely used in statistical mechanics and computer vision. In this paper, we use the Markov network to represent $P_{\mathcal {V}}$ and the microcircuit is the associated graph $\mathcal {G}$.

In a Bayesian network $\mathcal {B}=\{ \mathcal {G}, P\}$, the structure $\mathcal {G}$ is a directed acyclic graph. A parent node of $X_i$ is a node from which there exists a directed edge. The set of parent nodes of $X_i$ is denoted as $pa(X_i)$. The joint distribution of $\mathcal {V}$ can be factorized as $\prod _{X_i} P[X_i \vert pa(X_i)]$. Because of this factorization, the Bayesian network can represent the joint distribution in a compact fashion. The conditional independence in $\mathcal {G}$ can be examined by studying D-separation. The process of detecting $\mathcal {G}$ based on observed data is referred to as Bayesian network structure learning. A widely used Bayesian network structure learning approach is score-based structure learning. It defines a score function that measures how well the model fits the observed data; then finds the highest-scoring model. Relative to a Markov network, a Bayesian network has the potential to be used to detect causal relationships when the direction of information flow can be determined (Chen, 2021). Details of Bayesian network representation, D-separation, and structure learning are in  (Koller and Friedman, 2009). In this paper, we use a Bayesian network as a computational tool to facilitate Markov blanket discovery, instead of as a representation tool.

### Bayesian Coherence Analysis

BCA aims to learn a microcircuit $\mathcal {G}$ which is represented as a Markov network from a neural activity dataset $\mathbf {D}$. In a Markov network, the probability distribution has the form $P(x_1, ... , x_M) = \frac{1}{Z} \exp (- \sum _{c} V_c(\mathbf {x}_c)),$ where *c* is a clique (a fully connected subgraph) of $\mathcal {G}$, $\mathbf {x}_c = \{ x_i, i \in c \}$, $U = \sum _{c} V_c(\mathbf {x}_c)$ is the energy function and *Z* is the partition function. $P(x_1, ... , x_M)$ follows a Gibbs distribution with interaction potential $V_c(\mathbf {x}_c)$. In a pairwise Markov network, a clique contains up to two nodes. Pairwise Markov networks are widely used due to their computational efficiency (Hernández-Lemus, 2021). We adopt the pairwise Markov network representation in this paper. In a Markov network, the Markov blanket of node $X_i$ is the neighborhood of $X_i$. The Markov blanket of node $X_i$ is the set of nodes that are jointly most predictive of $X_i$. For node $X_i$, the edges between $X_i$ and nodes in $mb(X_i)$ are referred to as direct associations. For example, for a Markov network *A* – *B* – *C*, the direct association of node *C* is *B* – *C*. However, two nodes could be conditionally independent of each other but still associated. In this example, *A* and *C* are associated. A structure learning algorithm that does not consider conditional independence may generate a graph model including the edge *A* – *C*. This edge is referred to as indirect edges.

The neural activity data are often binary or continuous. For binary spike data, the state space $\Lambda$ is $\{0, 1\}$. The Ising model with fields can be used to represent coherence among binary nodes. In the Ising model with fields, $U = - (\sum _{i} r_i x_i + \sum _{i,j} w_{ij} x_i x_j)$, where $w_{ij}$ is the coupling coefficient and denotes the preference of nodes $X_i$ and $X_j$ to be in the same state. If $w_{ij} = 0$, then $X_i$ and $X_j$ are not connected. Let $\mathbf {r} = \{r_1, ..., r_M\}$ and $\mathbf {\Omega }$ be the matrix containing $w_{i,j}$. The matrix form of the energy function is $-(\mathbf {r}^T \mathbf {x} + \frac{1}{2} \mathbf {x}^T \mathbf {\Omega } \mathbf {x})$. For continuous data with the state space $\Lambda = \mathbb {R}$, a Gaussian Markov network can be used to model coherence. In this model, $P(\mathcal {V}) = \frac{1}{\sqrt{(2 \pi )^M} \vert \mathbf {\Sigma } \vert } \exp (-\frac{1}{2}(\mathbf {x}-\mathbf {\mu })^{T} \mathbf {\Sigma }^{-1}((\mathbf {x}-\mathbf {\mu }))$, where $\mathbf {\mu }$ is the mean vector and $\mathbf {\Sigma }$ is the variance-covariance matrix. Let $\mathbf {A} = -\mathbf {\Sigma }^{-1}$ and $\mathbf {b} = \mathbf {\mu }^T \mathbf {A}$. The matrix form of the energy function of a Gaussian Markov network is $-(\mathbf {b}^T \mathbf {x} + \frac{1}{2} \mathbf {x}^T \mathbf {A} \mathbf {x})$. The Ising model with fields and Gaussian Markov network have a unified representation.

For both the Ising model with fields and Gaussian Markov network, the neighborhood of node $X_i$ are nodes that are most predictive of $X_i$. Learning $\mathcal {G}$ (the structure of the network model) can be conducted in a node-by-node fashion. Therefore, Markov networks are a unified representation and learning framework for both binary and continuous neural activity data. The key task is to detect $mb(X_i)$. This is a challenging task when the dimensionality of $\mathcal {V}$ is high. For example, for a Gaussian Markov network, $\mathcal {G}$ can be detected based on partial correlation coefficients between nodes conditioned on all other nodes which can be obtained based on the negative inverse covariance matrix. Obtaining the negative inverse covariance matrix is computationally intensive for high-dimensional data.

**Fig. 1:**
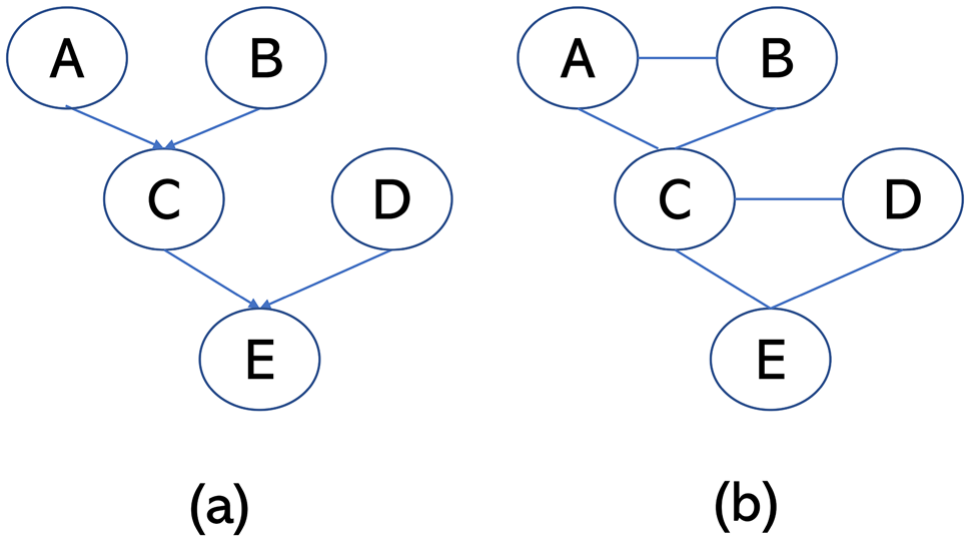
A Bayesian network (**a**) and the related Markov network (**b**)

When a probability distribution can be represented as a Markov network and Bayesian network, $mb(X_i)$ in these two models are identical. This is illustrated in Fig. 1. Figure 1(a) is a Bayesian network. In a Bayesian network, the Markov blanket of a node includes the parent node, the child node, and the parent set of child nodes. In Fig. 1(a), the Markov blanket of node *C* is $\{A, B, D, E\}$. We can convert the Bayesian network in Fig. 1(a) by moralization and obtain the associated Markov network which is depicted in Fig. 1(b). In Fig. 1(b), the neighborhood of *C* is $\{A, B, D, E\}$. The Markov blanket of *C* in these two probabilistic graphical models is identical. Based on this observation, $\mathcal {G}$ can be reconstructed by detecting the Markov blanket structure in a Bayesian network which is consistent with $\mathbf {D}$.

For high-dimensional data, directly learning the Markov blanket structure in a Bayesian network could be a daunting task. We have proposed a specific type of Bayesian network, called Bayesian Network Classifier with Inverse Tree structure (BNCIT), which supports efficient Markov blanket discovery for high-dimensional data (Chen & Herskovits, 2005a; b). Let $\mathcal {V} \setminus \mathbf {A}$ denote the set of nodes in $\mathcal {V}$ and not in $\mathbf {A}$. In BNCIT, to detect $mb(X_i)$, $X_i$ is set to be a leaf node and $\mathcal {V} \setminus \{ X_i \}$ are potential parent nodes of $X_i$. $mb^{*}(X_i)$, the detected Markov blanket, is a subset of $\mathcal {V} \setminus \{ X_i \}$ which can maximize a model fitness function $\Gamma (mb(X_i), \mathbf {D})$. That is,

1 $$\begin{aligned} mb^{*}(X_i) = \mathop {\mathrm {argmax}}_{mb} \Gamma (mb(X_i), \mathbf {D}). \end{aligned}$$

This optimization problem can be solved by the hill-climbing method. For binary data, the fitness function can be the Bayesian Dirichlet equivalent uniform (BDeu) score (Heckerman et al., 1995). For continuous data, the fitness function can be the Bayesian information criterion (BIC) (Schwarz, 1978). An important characteristic of these fitness functions is that they balance the likelihood and model complexity and tend to generate compact models. BNCIT is an approximate learning method to greatly improve learning speed. The Markov blanket of node $X_i$ discovered by BNCIT is guaranteed to be a subset of the ground truth Markov blanket (Chen & Herskovits, 2005a). BNCIT is fast and has very high specificity.

The BCA algorithm is listed in Algorithm 1. With BNCIT, we can learn the Markov blanket structure node by node. When $\mathcal {V}$ contains *M* nodes, this step generates *M* BNCIT models. Then we combine these models. If $X_i$ is in $mb^{*}(X_j)$ or $X_j$ is in $mb^{*}(X_i)$, the (*i*, *j*) element of $\mathcal {G}$ is 1 (nodes $X_i$ and $X_j$ are connected in the microcircuit); otherwise, it’s zero. The model combination step is important to improve sensitivity. 
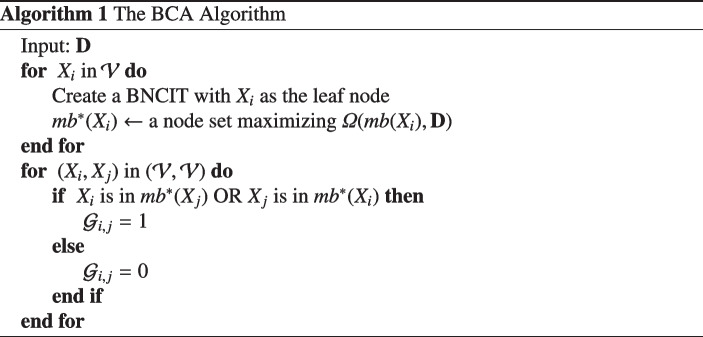


### Theoretical Analysis

The central assumption of BCA is as follows: if a probability distribution *P* can be represented by a Markov network $\mathcal {M}$ and a Bayesian network $\mathcal {B}$, and the Markov blanket structures of $\mathcal {M}$ and $\mathcal {B}$ are identical, then BCA can be used for approximate structure learning of $\mathcal {M}$.

Let *I*(*P*) be the set of conditional independence assertions that hold in *P*. Let $I(\mathcal {G})$ be the conditional independence encoded in the graph object $\mathcal {G}$. $\mathcal {G}$ is an I-map for *P* if $I(\mathcal {G}) \subseteq I(P)$. $\mathcal {G}$ is a minimal I-map for *P* if the removal of a single edge from $\mathcal {G}$ renders it not an I-map. $\mathcal {G}$ is a perfect map for *P* if $I(\mathcal {G}) = I(P)$. If *P* admits a Markov network which is a minimal I-map and also admits a Bayesian network which is a perfect map, then the Markov blanket structure in the Bayesian network is more compact. If *P* admits a Markov network which is a perfect map and also admits a Bayesian network which is a minimal I-map, then the Markov blanket structure in the Markov network is more compact.

1) If both $\mathcal {M}$ and $\mathcal {B}$ are perfect maps of *P*, then $I(\mathcal {M}) = I(\mathcal {B})$ and the Markov blanket structures of $\mathcal {M}$ and $\mathcal {B}$ are identical. Our assumption is satisfied. 2) When $\mathcal {M}$ is a minimal I-map and $\mathcal {B}$ is a perfect map, BCA can still detect the correct conditional independence structure in *P*. For this case, the Markov blanket structure detected by BCA is more compact than that generated by algorithms that directly learn the Markov network (such as the regression-based graph construction). 3) When $\mathcal {M}$ is a perfect map and $\mathcal {B}$ is a minimal I-map, the graph generated by BCA includes redundant links, relative to the graphs generated by algorithms that directly learn the Markov network. 4) When $\mathcal {M}$ is a minimal I-map and $\mathcal {B}$ is also a minimal I-map, it is not clear which graphical model is more compact. This depends on the characteristics of *P*.

Although it’s not guaranteed that *P* admits a Markov network or a Bayesian network which is a perfect map of *P*, a positive distribution *P* always admits a Markov network and a Bayesian network which are minimal I-maps of *P*. Details are in Sects. 3.4 and 4.4.3 of (Koller & Friedman, 2009). Therefore, in general, we can generate a probabilistic graphical model for the observed data. However, the generated model may not encode all conditional independence assertions in *P*.

Markov networks have been used to model single cell neuronal data (Makarenko et al., 1997; Schneidman et al., 2006; Ohiorhenuan et al., 2010). These studies center on coherence which is a symmetric relationship, instead of causal structures. Under some conditions such as a node order of causal relationship is given or intervention data are available, Bayesian network modeling can be used for causal discovery. In this setting, the edge direction has a causal interpretation. In BCA, we use a Bayesian network as a compact representation of a probability distribution. The edge direction in the generated model does not represent causality.

## Results

We assessed the performance of BCA in three studies: the 6-node model, biophysics-based simulation, and the Anterior Lateral Motor cortex (ALM) study. When a ground-truth graph $\mathcal {G}^{gt}$ is available, the performance of a microcircuit structure learning algorithm can be measured by comparing the estimated microcircuit $\mathcal {G}^{est}$ and $\mathcal {G}^{gt}$ and calculating the False Positive Rate (FPR) and True Positive Rate (TPR). FPR is the number of falsely identified links divided by the total number of empty links in $\mathcal {G}^{gt}$ where an empty link represents there is no edge between a node pair. TPR is the total number of correctly identified links divided by the total number of true edges in $\mathcal {G}^{gt}$. Sensitivity is equal to TPR and specificity is 1 - FPR. Both the 6-node model and biophysics-based simulation are simulation-based and have ground-truth graphs.

We implemented two comparison methods: correlation-based analysis and regression-based graph structure learning. In correlation-based analysis, we first calculated pairwise Spearman’s correlation coefficients. For $(X_i, X_j)$, if the associated p-value with the false discovery rate correction is smaller than a significance level, $\mathcal {E}^{est}_{i,j} = 1$; otherwise $\mathcal {E}^{est}_{i,j} = 0$. We used three significance levels: 0.05, 0.005, and 0.0005. The lower significance level represented a more conservative estimation. We used the method in  (van Borkulo et al., 2014) for regression-based graph construction. This method combines L1-regularized logistic regression with model selection based on the extended Bayesian information criterion. It generates a set of node-wise regression models and then combines them to form a graph model.

### The 6-node Model

**Fig. 2:**
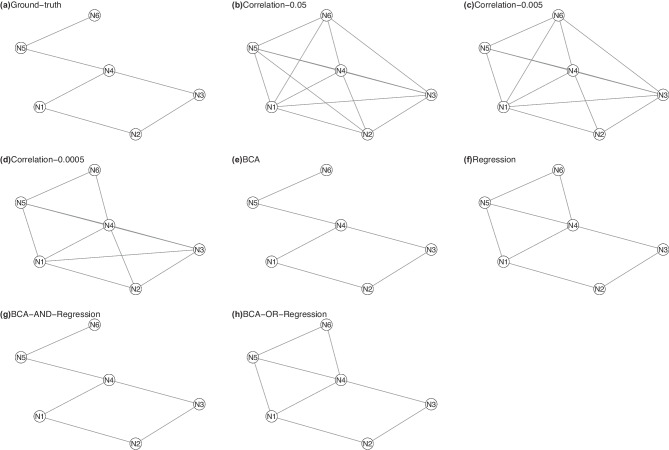
The microcircuits for 6-node model

We generated data for a microcircuit with 6 nodes. Each node was a neuron. The simulated data were sampled from an Ising model which is depicted in Fig. 2(a). The coupling coefficients were as follows: $w_{1,2}=0.5$, $w_{1,4}=0.5$, $w_{2,3}=0.5$, $w_{3,4}=0.5$, $w_{4,5}=-0.8$, and $w_{5,6}=0.9$. Temperature $\beta = 2$. The sample size was 5000. The sampling method was the Metropolis-Hastings algorithm.

**Table 1:** Structure learning for the 6-node model

Model	#edge	#empty	TP	FP	TPR	FPR
Correlation-0.05	6	9	6	8	1.00	0.89
Correlation-0.005	6	9	6	7	1.00	0.78
Correlation-0.0005	6	9	6	5	1.00	0.56
BCA	6	9	6	0	1.00	0.00
Regression	6	9	6	2	1.00	0.22
BCA-AND-Regression	6	9	6	0	1.00	0.00
BCA-OR-Regression	6	9	6	2	1.00	0.22

Figure 2(b)-(h) are network structures generated by different methods. Table 1 is TPRs and FPRs for different methods. In this table, the second column is the number of edges in $\mathcal {G}^{gt}$ and the third column is the number of empty edges in $\mathcal {G}^{gt}$. We also include two ensemble-based methods: BCA-AND-regression and BCA-OR-regression. In BCA-AND-regression, if a link exists in both the model generated by BCA and that of regression-based, it is in the final model. In BCA-OR-regression, if a link exists in either the model generated by BCA or that of regression-based, it is in the final model. BCA perfectly recovered the ground-truth structure. The regression-based method detected all edges, and added two spurious edges. The correlation-based analysis had a high FPR and detected many spurious edges. Relative to BCA, two ensemble-based methods achieved no performance gain. Although the data were sampled from a Markov network model, BCA which uses Bayesian network learning as the computation engine detected the ground-truth network structure with TPR=1 and FPR=0.

### Biophysics-based Simulation

**Fig. 3:**
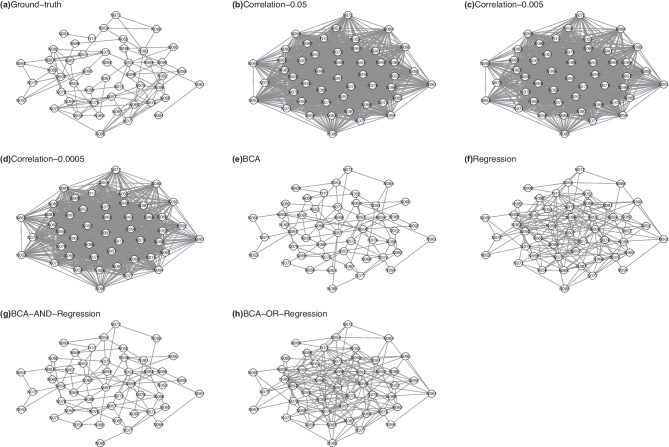
The microcircuits for biophysics-based simulation

We simulated 1200 samples for a feed-forward network with 100 integrate-and-fire neurons with additive noise. The neuron model was as follows: $\frac{dV}{dt} = \frac{V_{rest} - V}{\tau } + \sigma \times \epsilon \times \tau ^{-0.5}$, where *V* was the membrane potential and $V_{rest}$ was the resting potential. The neuron fired an action potential if $V > 1$. $\epsilon$ was a Gaussian random variable with mean 0 and standard deviation 1. $\sigma \times \epsilon \times \tau ^{-0.5}$ was the noise term and $\sigma$ controlled the noise level. $\sigma =0.2$. $\tau$ was the membrane time constant. There were two neural ensembles: groups A (50 neurons) and B (50 neurons). For group A, $\tau$ was a Gaussian random variable with mean 20 ms and standard deviation 5. For group B, $\tau = 100$ ms. The simulation sampling step was 5 ms. Neurons in group A were activated by an external stimulus. The external stimulus was randomly presented every 3 or 4 frames (15 or 20 ms). A neuron in group B was connected to two randomly selected neurons in group A. If a parent node fired, then the membrane potential of the target node increased by $a=0.8$. In the simulated data, one neuron in group B had no firing and was excluded from the analysis. The reference microcircuit $\mathcal {G}^{gt}$ is defined as follows: if neurons $X_i$ and $X_j$ in group B have at least one common parent node in group A, then $\mathcal {E}^{gt}_{i,j}=1$, otherwise $\mathcal {E}^{gt}_{i,j}=0$. This microcircuit for group B is depicted in Fig. 3(a). There were 98 edges in $\mathcal {G}^{gt}$.

This simulation is inspired by the studies demonstrating coupling spiking could be a consequence of shared presynaptic input (Shadlen & Newsome, 1998; Renart et al., 2010). Renart et al. investigated the relationship between coherence (measured by correlation) and shared excitatory inputs and found that the correlation between a neuron pair increased with increasing shared input fractions (Fig. 1b of  (Renart et al., 2010)). Our simulation focuses on examining the coherence of neurons in group B. The ground-truth connectivity matrix reflects shared inputs.

**Table 2:** Structure learning for biophysics-based simulation

Model	#edge	#empty	TP	FP	TPR	FPR
Correlation-0.05	98	1078	98	1028	1.00	0.95
Correlation-0.005	98	1078	98	966	1.00	0.90
Correlation-0.0005	98	1078	98	916	1.00	0.85
BCA	98	1078	63	30	0.64	0.03
Regression	98	1078	82	111	0.84	0.10
BCA-AND-Regression	98	1078	61	26	0.62	0.02
BCA-OR-Regression	98	1078	84	115	0.86	0.11

The results of structure learning are listed in Table 2 and the generated microcircuits are depicted in Fig. 3. Correlation-based analysis generated a dense network with many spurious edges (Fig. 3(b)-(d)). Other methods balanced true positives and false positives in different ways. Relative to BCA and regression-based methods, two ensemble-based methods (Fig. 3(g) and (h)) didn’t improve true positives and false positives significantly. True positives of BCA and regression-based were 63 and 82, while false positives of BCA and regression-based were 30 and 111. BCA missed extra 19 edges and eliminated 81 false positives. BCA was more balanced between sensitivity and specificity and had very low FPR.

### Anterior Lateral Motor Cortex

In the mouse, neurons in the anterior lateral motor cortex exhibit preparatory activity that predicts movements. The ALM in the mouse is a possible homologue of premotor cortex in primates. We reanalyzed two-photon calcium imaging data of ALM (Li et al., 2015). In this experiment, mice underwent a whisker-based object location discrimination task. A trial had three epochs: sample, delay, and response. A vertical pole was presented in the anterior or posterior position during the sample epoch. During the response epoch, mice reported the perceived pole position (posterior, lick right; anterior, lick left). Two-photon calcium imaging data were obtained for neurons in the left ALM which were labelled with GCaMP6s. The imaging depth was 410 $\mu m$. The number of observed neurons was 89 and the number of trials was 53. There were two trial types: lick left and lick right. Neural activity was measured by $\Delta F / F_0 = (F - F_0)/F_0$, where $F_0$ was the baseline fluorescence signal. For each trial, we used BCA to generate a microcircuit. This process resulted in 53 microcircuits.

**Fig. 4:**
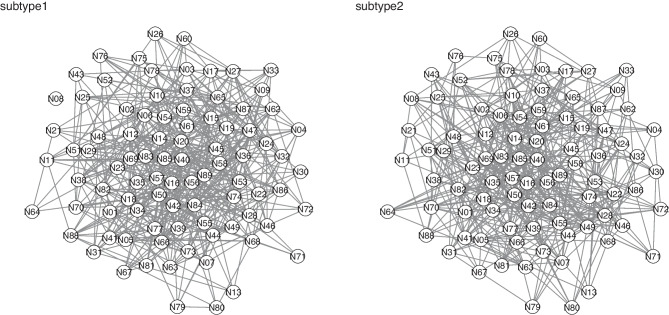
The representative microcircuits for subtypes 1 and 2. The microcircuits are generated based on calcium imaging data of the ALM study

**Fig. 5:**
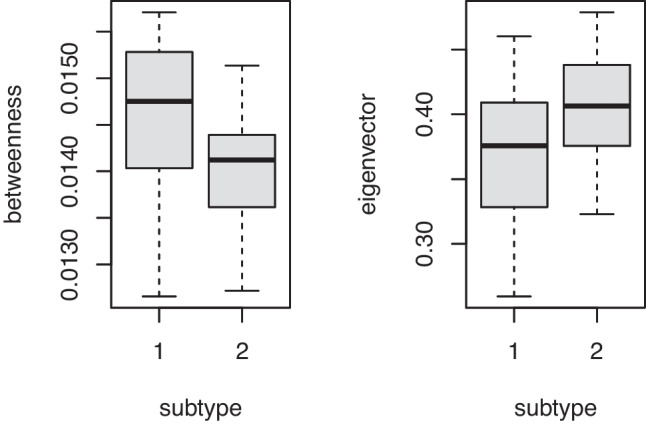
The boxplots of the betweenness and eigenvector centrality for subtypes 1 and 2 in the ALM study

We conducted subtype detection (Chen, 2021) to group the generated microcircuits into clusters. The similarity between $\mathcal {G}^a$ and $\mathcal {G}^b$ is the Sørensen-Dice coefficient of the adjacency matrix of $\mathcal {G}^a$ and that of $\mathcal {G}^b$. This similarity measure is in [0, 1] with 1 representing a perfect match and 0 representing no overlap. For these 53 trials, we generated a $53 \times 53$ similarity network, then used the multi-level modularity optimization algorithm to detect subtypes. For each subtype, we can select a representative graph that has the maximal similarity to other graphs in the same cluster. Two subtypes were detected (Fig. 4). The detected subtypes were highly predictive of trial types. Subtype 1 had 26 graphs for trial type ’left’ and 3 graphs for trial type ’right’ and subtype 2 had 1 graph for trial type ’left’ and 23 graphs for trial type ’right’ Fig. 5.

We can use graph-theoretic analysis for undirected graphs to understand the generated microcircuits. Node centrality scores quantify the importance of nodes based on their topological properties. A node centrality score can be calculated node-by-node. The graph-level node centrality score is the average across nodes. We used these graph-level node centrality metrics: betweenness centrality and eigenvector centrality (Fig. 5). Betweenness centrality measures how often a node is on the absolute shortest path between a node pair. Eigenvector centrality measures a node’s importance while considering the importance of the node’s neighbors. It is a generalization of the degree centrality and calculated based on the eigenvector of the graph adjacency matrix. For the ALM study, we compared these graph-level centrality scores across subtypes with two-sample t-tests (two-tailed). We found that subtypes 1 had a higher betweenness centrality score (p-value = 0.0036), and subtype 1 had a lower eigenvector centrality score (p-value = 0.0090). Nodes with high betweenness centrality often connect nodes in different network communities. The findings of graph-theoretic analysis suggest that microcircuits in subtype 1 prefer local computation and microcircuits in subtype 2 are more densely connected.

## Discussion

We propose BCA to learn the structure of a microcircuit from high-dimensional continuous or binary neural activity data. BCA treats structure learning as a node-wise Markov blanket discovery problem and utilizes Bayesian networks as computational tools to efficiently solve this problem. BCA incorporates the following major strengths. First, relative to correlation-based analysis which is one of the widely used methods for microcircuit construction, BCA can generate a compact model. Such compact models are parsimonious and facilitate downstream analysis such as clustering, differential analysis, and graph-based neural decoding (Chen & Lin, 2019). Second, BCA can handle high-dimensional data because it uses BNCIT for Markov blanket detection. BNCIT is a Bayesian network architecture to support approximate learning of Markov blanket with high specificity. Third, BCA is a unified structure learning framework for both continuous and binary neural activity data.

We compared BCA, correlation-based analysis, and regression-based analysis on a simulated dataset for a 6-node Ising model. BCA can perfectly recover the ground-truth structure while other methods have structure learning errors (Table 1). We assessed BCA’s performance on a biophysics-based simulation in which the reference microcircuit’s structure is determined by anatomical connectivity. Correlation-based analysis generates a very dense graph with many spurious edges. The microcircuit generated by BCA is well balanced in sensitivity and specificity. For the real-world ALM study, we constructed a microcircuit for each trial and obtained 53 microcircuits. We found that these microcircuits can be grouped into two subtypes that are highly predictive of trial types (lick left or lick right). This result demonstrates microcircuit structure learning with BCA can facilitate downstream analysis.

In this study, our goal is to learn the structure of a Markov network to describe the coordinated activity of neural firing in synchrony. The proposed method aims to address the limitations of the correlation-based analysis. We found that the proposed method generated more compact graphs than did the correlation-based analysis. First, the proposed method is not designed for causal discovery. Markov networks cannot model causal relationships. Figure 1 depicts the conditional independence structures, instead of causal relationships. Second, both Markov networks and Bayesian networks can be used to model conditional independence. For some conditional independence relations, it is possible that the Bayesian network representation is more compact. It is also possible that the Markov network representation is more compact (an example is in Sect. 4.1 of (Koller & Friedman, 2009)). We adopt the Markov network representation because the Bayesian network representation may not be unique. That is, an independence relation might admit many Bayesian network representations. Although the Markov blanket structures of these equivalent Bayesian networks are identical, the edge directions could be different. This might cause problems in downstream analysis such as graph clustering.

BCA is an approximate learning method. Its sensitivity could be improved by ensemble learning. However, ensemble learning methods often have much higher computation costs because they generate a set of models. We plan to assess the feasibility of combining BCA and ensemble learning under the Bayesian framework in our future work.

## Information Sharing Statement

The simulated data and the software package are freely available for academic purposes on request. The ALM dataset is is available at  (Li et al., 2015).

## Figures and Tables

**Fig. 6:**
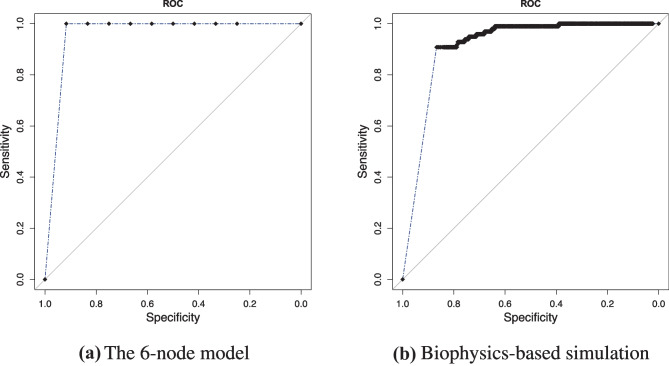
ROC curves for correlation-based analysis

## Appendix

In this experiment, we provided a comprehensive assessment of correlation-based analysis. Instead of setting a fixed significance level threshold $\alpha$, we treated $\alpha$ as a hyperparameter and generated a receiver operating characteristic (ROC) curve by varying $\alpha$. In the ROC curve, sensitivity is TPR and specificity is 1-FPR. The ROC curves for the 6-node model and the biophysics-based simulation are in Fig. 6(a) and (b). For the 6-node model, the operating point with the lowest FPR had FPR = 0.11 (one false-positive edge) and TPR = 1. If we lower $\alpha$ further, TPR would be 0. For BCA, FPR = 0 and TPR = 1. For the biophysics-based simulation, the operating point with the lowest FPR had FPR = 0.13 (143 false-positive edges) and TPR = 0.91 (89 correctly detected edges). For BCA, FPR = 0.03 and TPR = 0.64. Relative to correlation-based analysis with the lowest FPR operating point, BCA eliminated 113 false positives, with the cost of missing 26 true positives. If an application focuses on high TPR, then correlation-based analysis could be preferred. In this case, how to choose the threshold is critical. If an application focuses on generating compact models and achieving a balanced trade-off between TPR and FPR, BCA could be preferred.
