# Correction to: Bayesian Coherence Analysis for Microcircuit Structure Learning

**DOI:** 10.1007/s12021-022-09611-5

**Published:** 2022-10-13

**Authors:** Rong Chen

**Affiliations:** Department of Diagnostic Radiology and Nuclear Medicine, University of Maryland School of Medicine, 100 N Greene, Baltimore, 21201 MD USA

**Correction to: Neuroinformatics** **https://doi.org/10.1007/s12021-022-09608-0**

The original online version of this article was revised to update the data in Line 3 of Algorithm 1 from "$\mathbf{for}\;{X_i\;\mathrm{ in}\; S\;set\;\mathcal{V}}\;\mathbf{do}$." to "$\mathbf{for}\;{X_i\;\mathrm{in}\; \mathcal{V}}\;\mathbf{do}$."

Below is the correct presentation of Algorithm 1.
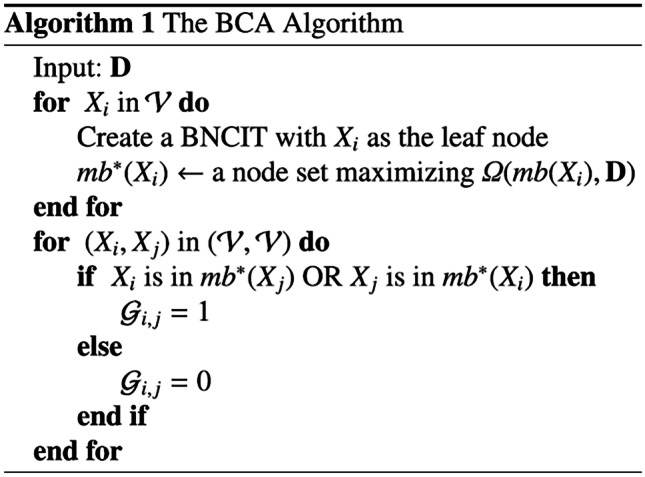


The original article has been corrected.

